# Design of CNS-Li_2_SiO_3_ Permeable Protective Coatings and Effects on Mortar Matrix

**DOI:** 10.3390/ma13071733

**Published:** 2020-04-08

**Authors:** Xu Li, Chonggen Pan, Dong Li, Jian Geng, Na Chen, Jingzi He, Shuhua Liu

**Affiliations:** 1 Department of Civil Engineering, Shanghai University, Shanghai 200444, China; 18764806631@163.com (X.L.); lidongbibo@163.com (D.L.); 2 Ningbo Institute of Technology, Zhejiang University, Ningbo 315100, China; gengjian@nit.net.cn; 3 Zhejiang YASHA Decoration Co., Ltd, Hangzhou 310008, China; 85chennana@163.com (N.C.); hejingzi@chinayasha.com (J.H.); 4 School of Water Resources and Hydropower Engineering, Wuhan University, Wuhan 430000, China; shliu@whu.edu.cn

**Keywords:** lithium silicate, colloidal nano-silica, protective coatings, mortar, microstructure

## Abstract

In this paper, we prepared permeable protective coatings composed of lithium silicate (Li_2_SiO_3_), where the coating was modified by colloidal nano-silica (CNS). Three levels of lithium silicate (i.e., 30 wt. %; 40 wt. %; 50 wt. %), sodium silicate (i.e., 5 wt. %; 10 wt. %; 15 wt. %), and surfactant (i.e., 0.05 wt. %; 0.1 wt. %; 0.15 wt. %) were involved in this study. An orthogonal experiment design selected the optimal proportion basedon thestrength and water absorption requirements of mortar. The effects of CNS-Li_2_SiO_3_ coating on the resistance to permeability of chloride ions and carbonation of specimens were also studied. The outcomes were interpreted using scanning electron microscopy (SEM), X-ray diffraction (XRD), and mercury intrusion porosimetry (MIP) techniques. The results showed that the optimum mix formulation consisted of 40 wt. % of lithium silicate, 10 wt. % of sodium silicate and 0.1 wt. % of surfactant within the mixtures investigated. Meanwhile, compared tothe control group, after the specimens were coated at 21 days curing age of mortar, the strength development, 48-h water absorption, resistance to chloride ions penetration, and carbonation of CNS-Li_2_SiO_3_ coated specimenswere improved. This could be attributed to the second hydration, leading to a reduction of the content of Ca(OH)_2_ and an increase of the amount of C–S–H gel within specimens. Thus, the microstructure of mortar matrix was improved after coated with CNS-Li_2_SiO_3_ permeable protective coatings.

## 1. Introduction

Cement-based material is a kind of heterogeneous material with many pores. Without any effective protection during servicelife, concrete structures are supposed to be affected by both physical actions and chemical erosion resulting from the environment. This process will lead to a degradation of the performance of cement-based materials. For instance, carbonation and chloride ions penetration will result in severe steel corrosion and damage to the concrete structure [1,2]. Meanwhile, environmental effects, such as wind-sand erosion or physical wear, will accelerate the destruction of the protective layer and subsequent corrosion of steel bars. At present, a series of protection measures for concrete structures are proposed, including various mineral admixtures, corrosion inhibitor incorporation, as well as anti-corrosion coatings for steel and concrete surfaces [3,4,5,6]. Pre-treatment for the concrete surfaces is an economical and effective approach among the types of protective measures [7]. Currently, the commonly used surface protective coatings are mainly organic materials such as silane impregnating materials, siloxane, acrylic acid, and epoxy resin materials [8,9]. However, organic coatings are prone to aging in service, and the protective effect is reduced significantly under elevated temperatures and ultraviolet radiation [10]. In addition to an improvement of concrete durability, the inorganic coatings also bring about a superior anti-aging performance in comparison to the organic counterparts, which broadens their scope of application [11,12].

At present, a series of investigations regarding the properties of water glass coatings have been performed. Pan et al. [15] studied the interaction between the concrete matrix and a combination of sodium fluorosilicate and water glass as surface treatment agents using microstructural analysis. The authors’ demonstrated that the combined treatment could generate more gel products because sodium fluorosilicate could accelerate the hardening of water glass. Moreover, both water glass and sodium fluorosilicate could react with cement hydrates, respectively. However, sodium silicate treatment may not be effective in terms of the prevention of chloride ions into the concrete, where the researchers used sodium silicate, silane, and siloxane to treat concrete specimens and placed them into seawater for dry/wet cycles for up to one year [16]. The combined treatment with sodium silicate and nano silica has smaller effects on specimens than ethyl silicatetreatment, according to several indicators such as resistance to wear, chloride ions penetration, and carbonation [17]. Given the studies above, it was found that the combination of sodium silicate and curing agent may not be a good choice for the treatment of the concrete surface. Its impact on the durability improvement of concrete is not significant, because the content of new C-S-H gels is not enough to fill the most harmful capillary pores. This is pronounced when it comes to the resistance of chloride ions penetration and carbonation. Meanwhile, studies regarding the involvement of lithium silicate protective coatings of concrete are scarce, albeit there are still some studies such as References [18,19] that adopted lithium silicate composite in protective coatings. However, these studies were focused on the performance of the concrete surface, as well as its microstructures. They failed to investigate the effects of Li_2_SiO_3_ coatings on the resistance of chloride ions and carbonation of specimens, and its mechanism of action is still not clarified. In addition, due to the small particle size and high surface activity of nanoparticles, it is difficult to disperse evenly into organic media. Still, it is compatible with inorganic media, and it has a large penetration depth in cement-based materials than Li^+^ (Li_2_SiO_3_). Moreover, nano-SiO_2_ particles can improve the mechanical properties of cement-based materials, to accelerate the hydration process, and densify the microstructures of cement-based materials [20,21]. Nanoparticles can enhance the contact angles on coated concrete and reduce water absorption by increasing the degree of surface roughness of organic coatings [22]. Still, the dispersion of nanoparticles in organic medium presents a difficulty, by easily producing defects within polymeric film coatings [23]. Meanwhile, studies on the influence of nano-SiO_2_ particles on lithium silicate based inorganic coatings of concrete are also scarce.

Water glass is one of the materials applied in inorganic coatings. It can be divided into sodium silicate, potassium silicate, and lithium silicate [13]. It is reported [14] that water glass inorganic coatings are capable of penetrating the deeper section of concrete, and some new phases form within the concrete matrix as a result of physical and chemical reactions, filling into the capillary pores in concrete. Thus, the waterproof performance and impermeability of concrete can be improved due to the prevention of external corrosive media, which leads to an improvement of concrete durability. Moreover, due to a smaller radius of Li^+^ and a higher modulus of Li_2_SiO_3_ compared to Na^+^ (Na_2_SiO_3_) and K^+^ (K_2_SiO_3_), as expected, Li_2_SiO_3_ outperforms Na_2_SiO_3_ and K_2_SiO_3_ solutions given their permeability into the deeper section of concrete and self-curing ability. 

Given the above, this study selected the optimal proportion of Li_2_SiO_3_, Na_2_SiO_3,_ and fatty alcohol polyoxyethylene ether nonionic surfactant in the coating using an orthogonal experiment. It was aimed at the durability of Li_2_SiO_3_ coating on the surface of cement-based materials, including resistance to chloride ions penetration and carbonation. Therefore, nano SiO_2_ sol was incorporated into an inorganic composite with Li_2_SiO_3_ coating to prepare CNS-Li_2_SiO_3_ permeable protective coating. The effects of the coating on the strength, water absorption, resistance to chloride ions penetration, and carbonization of the mortar matrix were studied. The effects of the coatings on the surface microstructure of cement-based materials were analyzed using the SEM, XRD, and MIP techniques. Finally, the mechanism of action was described in terms of the nano-modified coating on the mortar surface.

## 2. Materials and Methods

### 2.1. Raw Materials

The cement used was PO42.5 Portland cement from Sanshi Company (Zhejiang, China). The main oxide compositions of the cement are summarized in Table 1. The sand was ISO standard sand from Aisiou Standard Sand Company (Xiamen, China). Mortar specimens were prepared according to the mix ratio C(cement): S(sand): W (water)= 1:3:0.5 by mass. The raw materials of the coatings consisted of: (1) lithium silicate solution from the Lvsen Chemical Company (Linyi, China) (Chemical formula:Li_2_O∙mSiO_2_; Modulus: 4.8; Solid content: 23%); (2) sodium silicate solution from the Dongyue Chemical Company (Zibo, Shandong, China) (Chemical formula:Na_2_O∙mSiO_2_; Modulus: 3.3; Solid content: 34%); (3) alkaline nano SiO_2_ sol from the Haiwan Company (Qingdao, China) (Chemical formula:mSiO_2_∙nH_2_O; SiO_2_ content: 30.0%; Particle size: 10–20 nm); (4) fatty alcohol polyoxyethylene ether nonionic surfactant (PAE) from Yiqun Chemical Company (Linyi, China); (5) silicone defoaming agent from the Lvsen Chemical Company (Linyi, China); (6) polyethylene glycol PEG300 dispersant from the GHTECH Company (Shantou, Guangdong, China); and (7) AMP-95pH regulator from the Qingchun Chemical Company (Jinan, China).

### 2.2. Orthogonal Experimental Design

To reduce the experiment numbers, we designed an orthogonal experiment with three factors and three levels, as shown in Table 2. Factor A was lithium silicate, with three levels by mass of the coatings: A1 (30 wt. %), A2 (40 wt. %), and A3 (50 wt. %). Factor B was sodium silicate, with three levels: B1 (5 wt. %), B2 (10 wt. %), and B3 (15 wt. %). Factor C was surfactant, with three levels: C1 (0.05 wt. %), C2 (0.1 wt. %), and C3 (0.15 wt. %). The mortar surface was treated with nine kinds of coatings (Table 2) at a curing age of 7, 14, and 21 days, respectively. The optimal mix formulation of the coating was selected, given the strength and water absorption of the coated specimens.

### 2.3. Process of Coating Preparation

According to the mix formulation, the procedures of coating preparation were as follows: (1) different amounts of deionized water, pH regulator, and dispersant were added into the magnetic agitator, and they were stirred at high speed of 1500 r/min for 30 min. (2) Lithium silicate/sodium silicates sol of different proportions were mixed with the previously prepared solution, and they were stirred for another 30 min. (3) Various amounts of surfactants were then added to the solution for another 3 h. (4) The defoamer was added to the solution and stirred with a reduced mixing speed of <500 r/min to a foam-free state. Finally, the solution was kept sealed. (5) After 24 h, the nano-SiO_2_ sol was blended with the prepared coatings drop by drop according to a mass ratio of 3:10, stirred at a speed of 1500 r/min until the solution was clear and transparent, and the CNS-Li_2_SiO_3_ permeable protective coatings were prepared. They were sealed and reserved. (6) At a curing age of 6, 13, and 20 days, the specimens were moved from the standard curing room, polished with 150-mesh sandpaper, and dried for 1 day at room temperature. Then the mortar surface was coated with a hairbrush. After the surface was dried again, the specimens were preserved in a curing room (T = 20 °C and RH = 60%) until a curing age of 28 days of mortar.

### 2.4. Methods

#### 2.4.1. Compressive Strength

Compressive strength was tested with three specimens for each coating. The size of the specimen was 40 mm × 40 mm × 160 mm. The loading rate of the instrument (hydraulic pressure testing machine, SYE-300A, Cangzhou, China) was 2400 ± 200 N/s. The testing procedure was consistent with GB/T 17671-1999 (method of testing cement-determination of strength) [24].

#### 2.4.2. Water Absorption

The water absorption after 48 h in the specimens was used as the index of the water absorption test. The index was averaged from the results of the three specimens, where the size was 70 mm × 70 mm × 70 mm. The bottom surface of the specimens was coated as the experimental surface, and the rest of the surfaces were sealed with epoxy resin. Before the experiment, the specimens were placed into the drying box at 78 °C for 48 h. Afterward, they were placed (with the coated surfaces face down) into a water tank with two steel bars at the bottom, and the specimens were immersed in water at the height of 35 mm until the experiment finished. The experiment was conducted in line with JGJ/T 70-2009 (standard test method for basic properties of construction mortar) [25]. The 48-h water absorption of the specimens was calculated using Formula (1):

(1) $$W_{x}=\frac{m_{1}-m_{0}}{m_{0}}$$

where: *W_x_* is the 48-h water absorption of the mortar (%), *m*_1_ is the mass of the specimens after water is absorbed (g), and *m*_0_ is the mass of the dry specimen (g).

#### 2.4.3. Resistance to Chloride Ions Permeability

The size of the specimens was 40 mm × 40 mm × 160 mm. The surfaces of the specimens were treated at the curing age of 21 days, and the methods of surface treatment are referred to in Subchapter 2.4.2. The specimens were soaked in a NaCl solution with a concentration of 3.5 wt. %, and then they were taken out after soaking for up to 28 days. The penetration depth of free chlorine ions into the mortar was determined usingthe silver nitrate (AgNO_3_) spray technique as shown in Reference [26]. The withdrawn specimens were split in half, and the surfaces of the freshly split specimens were sprayed with a 0.1 M AgNO_3_ solution. The presence of free chlorides was indicated by the formation of a white precipitate of silver chloride (AgCl). Meanwhile, the absence of free chlorides was indicated by brown silver hydroxide (AgOH). The penetration depth of free chlorine ions was determined by taking linear measurements from the edge of the specimen up to the color change boundary, and every measurement was taken at an interval of 5 mm.

#### 2.4.4. Resistance to Carbonation 

The size of the specimens was 40 mm × 40 mm × 160 mm.The accelerated carbonation experiment was conducted according to GB/T50082-2009 (standard test method for long-term performance and durability of ordinary concrete) [27]. Only one surface was coated as the experimental surface, and the rest surfaces were sealed with epoxy resin.The carbonation depth was measured for 28 days, and each result was averaged from the results of three specimens. Each specimen was measured for three fracture surfaces. Every measurement was taken at an interval of 5 mm.

#### 2.4.5. SEM Analyses

The coated and uncoated chunks were hydrated with ethanol at the curing age of 28 days. The microstructures of the specimens were analyzed through a Zeiss Evo-18 scanning electron microscope (SEM) (Oberkochen, Germany). Before the SEM, the chunks were also coated with gold to increase their conductivity.

#### 2.4.6. XRD Analyses

Some cement particles extracted from the coated and uncoated parts in the specimens were ended with hydration with ethanol and further ground into a powder that could pass through a 150 µm sieve before XRD. The change of cement hydration products within the uncoated and coated parts was tested using a D8 Advance x-ray diffraction (XRD) (Karlsruhe, Germany).The scan rate was the standard rate, and the scan angle was from 5 to 90°. 

#### 2.4.7. MIP Analyses

The chunks were taken from a 3 mm thick surface layer of mortar matrix specimens after strength was measured.Then they were crushed, and the chunks with a size of 3–5 mm were taken for the MIP test. Afterward, a vacuum drying box at 105 °C was used to dry the specimens to a constant weight, and then they were placed into a sealed bag. The pore structures of the specimens were investigated using an AutoPore IV 95 automatic mercury injection instrument (Norcross, America), with a maximum mercury pressure of 60,000 PSI.

## 3. Results and Discussions

### 3.1. Selection of the Optimal Mix Formulation Concerning Coating Preparation 

Number 1–9 coatings were prepared according to the mix formulation in Table 2. The mortar specimens were coated at a curing age of 7, 14, and 21 days. The compressive strength and water absorption of the specimens are recorded in Table 3. The results were used in the selection of the optimal mix formulation in terms of coating preparation. 

**Figure 1:**
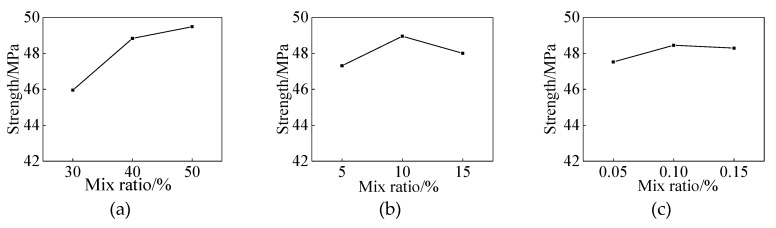
Impact of each factor on compressive strength, (**a**) Lithium silicate; (**b**) Sodium silicate; (**c**) Surfactant.

**Figure 2:**
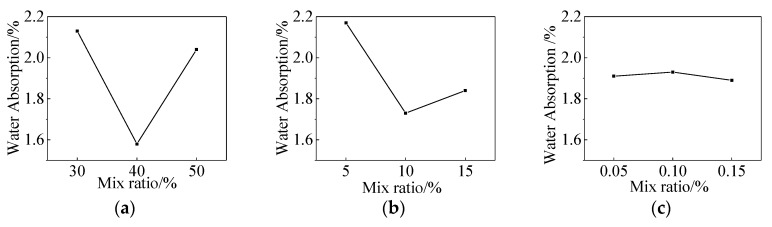
Impact of each factor on 48-h water absorption, (**a**) Lithium silicate; (**b**) Sodium silicate; (**c**) Surfactant.

The compressive strength and 48-h water absorption of the uncoated specimens were 42.87 MPa and 3.28%, respectively. As shown in Table 3, when the specimens were coated at the curing age of 7 days, the influence of the coating on the compressive strength and water absorption of the specimens could be ignored. However, its impactbecame more evident with the increase of the curing age of the mortar. For instance, the maximum compressive strength of the specimens revealed 49.95 MPa with the lowest water absorption, reaching 1.33% when the specimens were coated at the curing age of 21 days. Meanwhile, the range analysis of experimental results obtained from treated specimens at the curing age of 21 days is shown in Table 4. The analysis of variance (ANOVA) for compressive strength and water absorption of the coated specimens is shown in Table 5. The effects of each factor on compressive strength and 48-h water absorption are shown in Figure 1 and Figure 2. The figures demonstrate that these two indicators of specimens, A3B2C2 and A2B2C3, are the optimal mix formulation during coating preparation. As shown in Table 5, factor C was considered a non-significant impact on compressive strength and water absorption, and it was pooled. The F statistic indicated that factor A had the most significant impact on the strength and water absorption of coated specimens.This was followed by factor B. The compressive strength and water absorption of the coated specimens were found to be dominated by two different factors, namely lithium silicateand sodium silicate. The optimal mixing proportion of the three factors in the coating consisted of 40 wt. % of lithium silicate, 10 wt. % of sodium silicate, and 0.1 wt. % of surfactant.

### 3.2. Effects of CNS-Li_2_SiO_3_Coating on the Compressive Strength and Water Absorption of the Specimens

**Figure 3:**
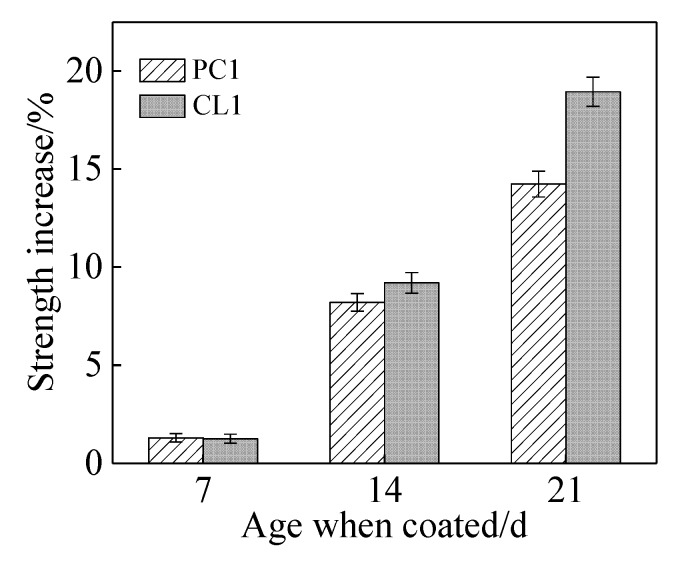
The rate of compressive strength in coated specimens.

**Figure 4:**
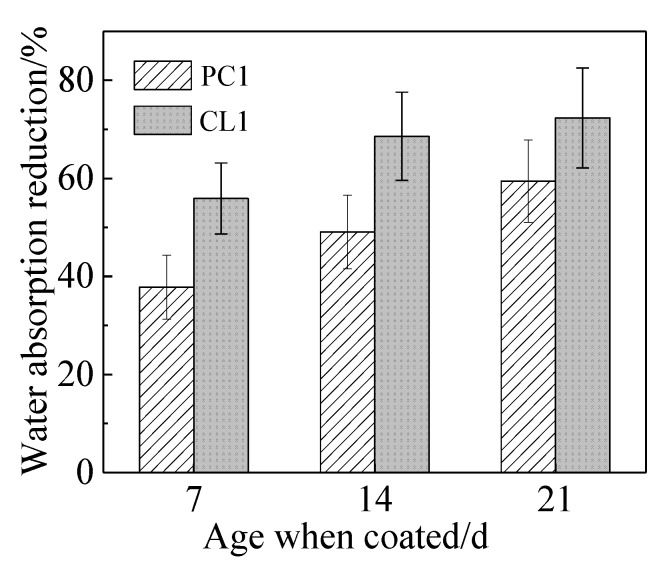
Rate of 48-hwater absorptionin specimens.

Based on the results of the orthogonal experiment, a coating named protective coating (PC1) was prepared according to the optimal mixing ratio. The nano-SiO_2_ modified lithium silicate coatingwas named CNS-Li_2_SiO_3_ coating (CL1). When the specimens were coated using PC1 and CL1 coating at the age of 7, 14, and 21 days of mortar, the improvement of compressive strength and reduction of 48-h water absorption of the specimens are presented in Figure 3 and Figure 4. They were compared to the uncoated specimens. Irrespective of coating materials, the influence of the coating on mortar became more obvious in terms of the compressive strength and the water absorption of specimens over the curing age of mortar. For instance, when the specimens were coated with CL1 coating at the curing age of 21 days, an improvement in compressive strength (i.e., 19.8%) and a reduction in water absorption (i.e., 72.3%) was achieved in the coated specimens compared to the uncoated specimens. The functional components of coating, namely lithium silicate and sodium silicate, in PC1 coating, and nano-SiO_2_, in CL1 coating, could react with one of the hydration phases, Ca(OH)_2_, within cement system. C–S–H gels were formed, which contributed to the binding capacity for the cementitious system as a consequence of filling capillary pores within specimens [28]. This is elaborated in Section 3.5. Since the content of Ca(OH)_2_ in later cement hydration ages was higher than that in early age, that is the reason why pronounced effects were observed when specimens were coated with two coatings at a later age. On the one hand, the nano-SiO_2_ can permeate into the capillary pores of the mortar as a filler to improve the mortar strength.On the other hand, there are adhesion effects between calcium ions and nano-SiO_2_ that can affect nucleation sites [29]. The pores in the mortar surface layer could be filled by the C–S–H gels, so the compressive strength and water absorption of specimens would improve.

### 3.3. Effects of CNS-Li_2_SiO_3_ Coating on Chloride ion Penetration Resistance of THE Specimens

**Figure 5:**
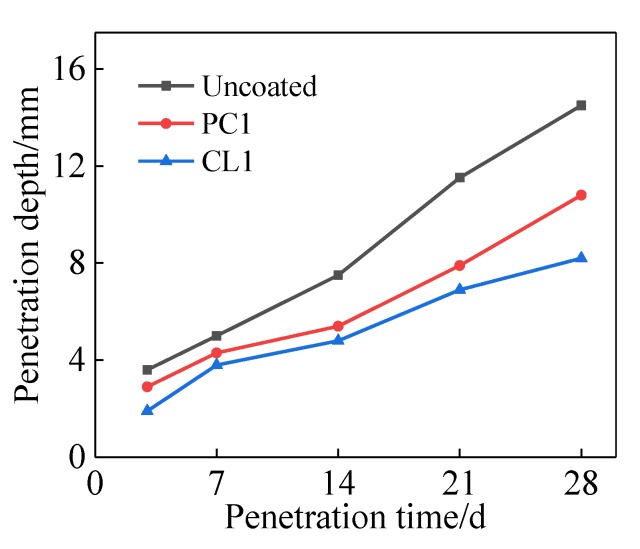
Impact of the coatings on chlorideion penetration depth of specimens.

Figure 5 shows the chloride ion penetration depth of coated and uncoated specimens. The coated specimens were treated with PC1 and CL1 coatings at the age of 21 days of mortar. The effect of PC1 coating on the resistance to chloride ion penetration within specimens was not as pronounced as CL1 coating. This was reflected by the 28-day chloride ion penetration depth (i.e., 8.2 mm versus 10.8 mm). The findings were in line with a study performed by Franzoni et al. [17]. They reported that chloride ion penetration depth of specimens treated by silica nanoparticles and sodium silicate sol could be reduced by 25% and 40%, respectively. The size of capillary pores within specimens becomes smaller after the coating treatment, which candelay the migration of free chlorine ions into the deeper section of the specimens [30]. Given increases in the amount of C–S–H gels in the surface layer of the mortar, several chlorine ions were adsorbed by newly formed C–S–H gels.

### 3.4. Effects of CNS-Li_2_SiO_3_ Coating on the Carbonation Resistance of Specimens

**Figure 6:**
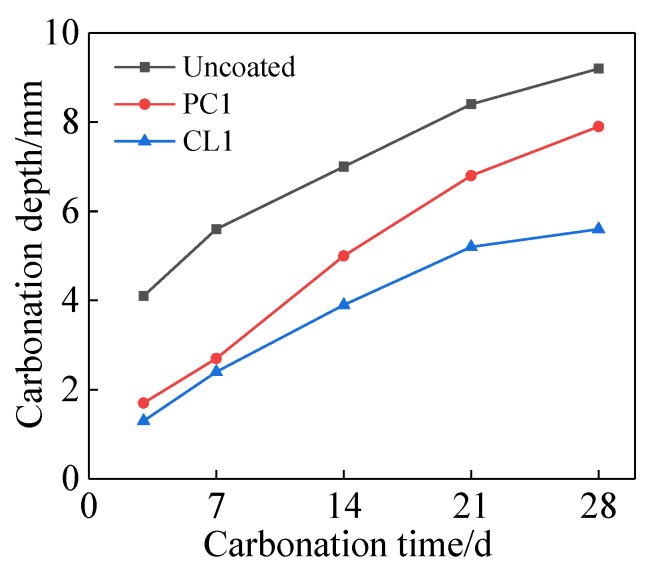
Effect of coatings onthe carbonation depth of specimens.

Figure 6 shows the influence of the coating on the carbonation depth of specimens. It shows that the carbonation resistance was improved after the specimens were treated with PC1 and CL1 coatings, as expected. Meanwhile, PC1 coating only performed well in the early-age carbonation resistance of specimens. Still, it failed to protect the mortar further at a later age, reflected by a small reduction of the 28-day carbonation depth (i.e., 14.1%) compared to the uncoated specimens. The trend was also confirmed in Reference [17]. In contrast, a different scenario was seen in CL1 coated specimens, which revealed long-lasting protection of specimens given the carbonation resistance of the specimens (i.e., 39.1% smaller after 28 days than the control). This was proven by a slowing down of carbonation depth increment in specimens treated with CL1 coating. As mentioned, CL1 coating could greatly reduce the surface porosity of the mortar, which inhibits the ingression of CO_2_. Moreover, due to the reaction between CO_2_ and Ca(OH)_2_ and their reaction product, CaCO_3_ can fill the capillary pores within the specimens. Thus, a more compact carbonation layer formed on the surface of specimens that could further reduce the carbonation rate. Moreover, lithium silicate is used as one of the raw materials of sorbents for CO_2_ so that it can also reduce the carbonation rate [31].

### 3.5. Effects of CNS-Li_2_SiO_3_ Coatings on the Microstructuresand Pore Structures of Specimens

**Figure 7:**
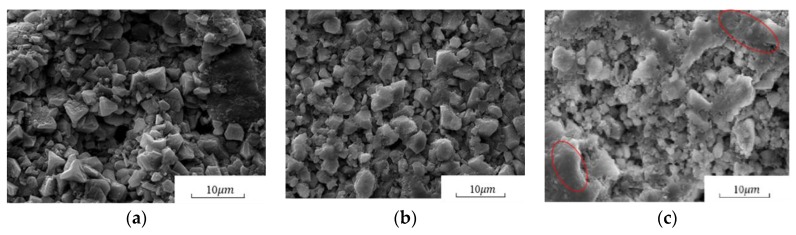
Effect of coatings on the SEM images of hardened cement paste: (**a**) Uncoated specimens; (**b**) PC1; (**c**) CL1.

Figure 7 shows the SEM images of the coated and uncoated specimens at a 2000× magnification. There were many pores on the surface of the uncoated specimens, and the crystal structure of cement hydration products was irregular, indicating a low hydration degree of cement. However, the crystal structure became regular after being treated with PC1 coating due to the increase in C–S–H gels amount. Meanwhile, owing to the coacervate of nano-SiO_2_, the original grains began to disappear and the crystals became more interconnected with each other. Thus, a denser microstructure of specimens was formed after being coated with CL1 coating.

**Figure 8:**
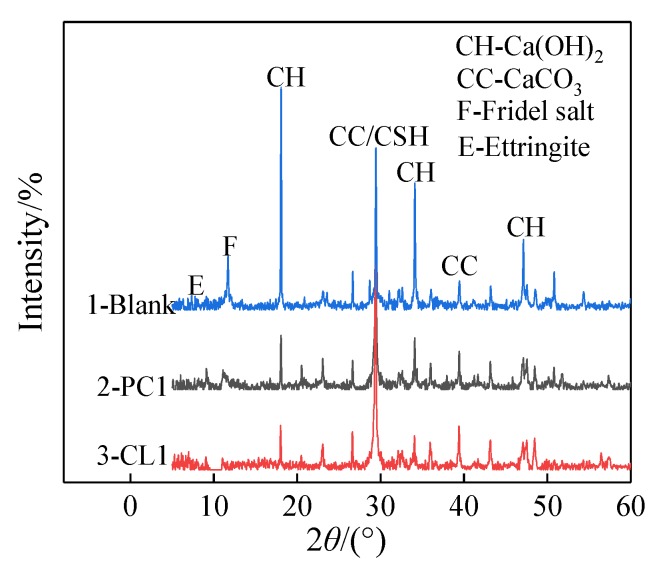
Effect of coatings on XRD patterns of cement hydration products.

**Figure 9:**
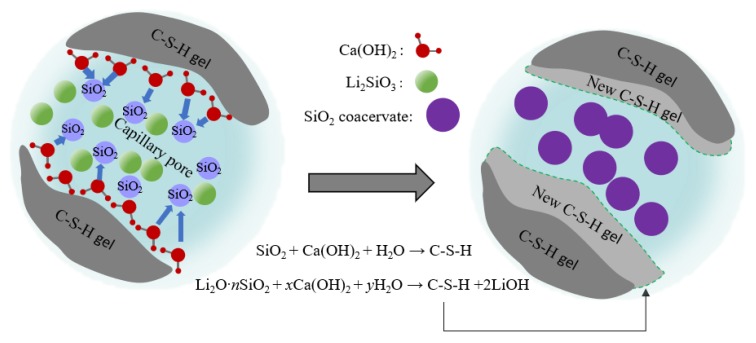
Mechanism of coating and cement-based materials.

Figure 8 shows the XRD patterns of cement hydration products, and the main hydration phase of Ca(OH)_2_ and C–S–H gel can be identified from the patterns. The presence of Ca(OH)_2_ failed to contribute to the strength development of specimens since it was a layered crystal prone to slip and quite weak in strength and bond after external erosion [32]. However, after PC1 and CL1 coatings were applied, as shown in Figure 8, the intensities of Ca(OH)_2_ peaks became smaller, accompanied by an increase of C–S–H gel peak intensities. The reaction mechanism between coating materials and specimens is shown in Figure 9.The figure shows that the Li_2_SiO_3_ and SiO_2_ in coatings can react with the Ca(OH)_2_ in the cement system due to the secondary hydration, and additional C–S–H gels were formed. Meanwhile, as the nucleation sites, SiO_2_ nanoparticles were also capable of gathering Ca(OH)_2_ on the surface of the specimens, and their large surface energy further reduced the orientation degree of Ca(OH)_2_ [33]. They also accelerated the formation of C–S–H gels. Moreover, nano-SiO_2_ particles coacervate can also act as inert fillers within pores between particles, which leads to a more compact structure, as shown in Figure 7c.

**Figure 10:**
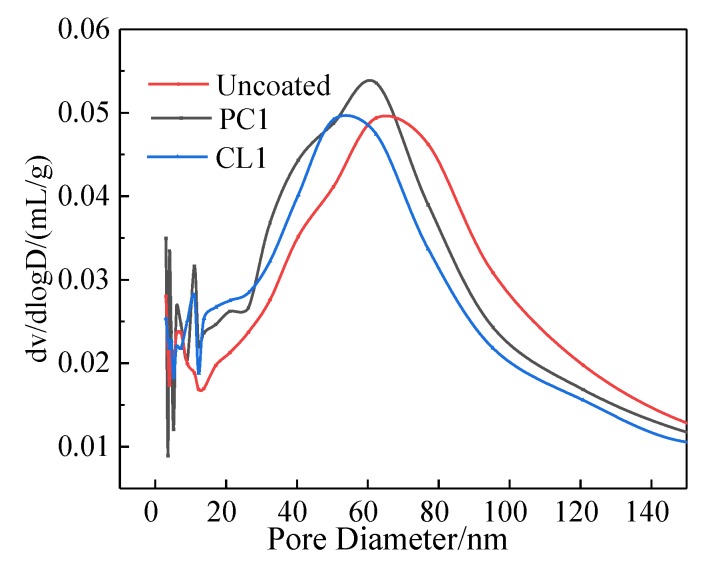
Effect of coatings on the pore sizedistribution of specimens.

It is known that the pore size distribution of cement-based materials varies, and Wu et al. [34] divided the pore size into three categories: (1) Harmless pores (<20 nm); (2) Less harmful pores (20~100 nm); (3) Harmful pores (>100 nm). Figure 10 shows the size distribution of 3~150 nm pores in the surface interface layer of uncoated and coated specimens. The most probable pore size of the uncoated specimen was about 70 nm, while after PC1 and CL1 coatings treatment, the most probable pore size became smaller, reaching 60 nm and 50 nm, respectively. The application of PC1 coating led to an increment of harmless pores and less harmful pores volume by 5.15% and 15.7%, respectively, within the interface layer specimens. The diameter of less harmful pores ranged between 20 and 60 nm, whereas the volume of harmful pores decreased by 10.2%. In terms of specimens with a CL1 coating, an increase of 16.32% in harmless pores and 8.4% in less harmful pores volume was achieved. The harmful pores also reduced by 4.7%.

## 4. Conclusions

In summary, the nano-modified CNS-Li_2_SiO_3_ permeable protective coating designed in this study improved the compressive strength, water absorption, resistance to chloride ions penetration. The carbonation of specimens and the microstructures of the interface layer within specimens were more compacted and denser. Specific conclusions were as follows:

(1) The optimal mix formulation consisted of 40 wt. % of lithium silicate, 10 wt. % of sodium silicate, and 0.1 wt. % of surfactant in the preparation of Li_2_SiO_3_ coatings, obtained by an orthogonal experiment design.
(2) Compared with an early age, the specimens treated with CNS-Li_2_SiO_3_coatingat a later age of mortar were more effective, citing compressive strength and water absorption of the specimens. This was due to the abundance of Ca(OH)_2_ present within specimens at a later age.
(3) CNS-Li_2_SiO_3_ coatings favored a compressive strength increase (i.e., 19.8%), a reduction of 48-h water absorption (i.e., 72.3%), a lower chloride ion penetration depth(i.e.,44.2%), and a smaller carbonation depth(i.e., 39.1%) of specimens compared touncoated specimens. This could be attributed to the porosity reduction and denser microstructures formed owing to the inter fill of nano-SiO_2_ and the formation of additional C–S–H gels resulting from secondary hydration. Meanwhile, the new C–S–H gels formation was confirmed by an increment of harmless pores (i.e., 16.32%), as well as a reduction of harmful pores (i.e., 4.7%) within coated specimens in comparison to their uncoated counterparts.

## Figures and Tables

**Table 1:** Main oxide compositions of cement.

Oxide	SiO_2_	CaO	Fe_2_O_3_	Al_2_O_3_	MgO	f-CaO	LOI (Loss on Ignition)
**Content (%)**	22.56	61.96	3.89	5.56	1.93	0.5	1.64

**Table 2:** Absolute values of the variables (Li_2_SiO_3_, Na_2_SiO_3,_ and PAE) and the orthogonal array for L_9_ (3^4).

Sample	Factors, Their Code (Levels) and Absolute Values					
	Li_2_SiO_3_ (%)		Na_2_SiO_3_ (%)		PAE (%)	
	Coded	Values	Coded	Values	Coded	Values
**1**	A1	30	B1	5	C1	0.05
**2**	A1	30	B2	10	C2	0.1
**3**	A1	30	B3	15	C3	0.15
**4**	A2	40	B1	5	C2	0.1
**5**	A2	40	B2	10	C3	0.15
**6**	A2	40	B3	15	C1	0.05
**7**	A3	50	B1	5	C3	0.15
**8**	A3	50	B2	10	C1	0.05
**9**	A3	50	B3	15	C2	0.1

**Table 3:** Experimental results of the compressive strength and water absorption of mortar.

Samples	Compressive Strength (MPa)			Water Absorption (%)		
	7d	14d	21d	7d	14d	21d
1	42.07 ± 2.04	42.12 ± 1.69	44.67 ± 2.25	3.06 ± 0.31	2.58 ± 0.26	2.36 ± 0.25
2	42.49 ± 1.69	44.87 ± 1.58	47.92 ± 2.35	2.60 ± 0.27	1.90 ± 0.13	1.88 ± 0.20
3	42.10 ± 2.15	44.54 ± 2.22	45.27 ± 2.89	2.62 ± 0.22	1.87 ± 0.21	2.14 ± 0.12
4	42.83 ± 2.39	45.72 ± 2.26	47.62 ± 2.66	2.79 ± 0.34	2.02 ± 0.25	1.96 ± 0.24
5	42.87 ± 2.11	45.84 ± 2.15	49.95 ± 2.72	2.04 ± 0.17	1.67 ± 0.23	1.33 ± 0.10
6	42.56 ± 2.05	46.36 ± 2.17	48.92 ± 2.42	2.46 ± 0.12	1.59 ± 0.29	1.44 ± 0.21
7	42.96 ± 1.88	45.57 ± 2.15	49.65 ± 1.95	2.58 ± 0.15	2.24 ± 0.26	2.19 ± 0.15
8	43.09 ± 1.95	46.33 ± 2.39	48.98 ± 2.12	2.59 ± 0.25	2.26 ± 0.21	1.98 ± 0.25
9	43.16 ± 1.39	46.42 ± 2.02	49.82 ± 2.55	2.68 ± 0.24	2.16 ± 0.22	1.94 ± 0.19

**Table 4:** Analysis of each factor.

No.	Compressive Strength (MPa)			Water Absorption(%)		
	A	B	C	A	B	C
**K_1_**	137.86	141.94	142.57	6.38	6.51	5.77
**K_2_**	146.49	146.85	145.36	4.73	5.19	5.79
**K_3_**	148. 45	144.01	144.87	6.11	5.52	5.66
**k_1_**	45.95	47.31	47.52	2.13	2.17	1.92
**k_2_**	48.83	48.95	48.45	1.58	1.73	1.93
**k_3_**	49.48	48.00	48.29	2.04	1.84	1.89
**R**	3.53	1.38	0.93	0.55	0.44	0.04
**Better Level**	A3	B2	C2	A2	B2	C3
**Major Factor**	A > B > C			A > B > C		

**Table 5:** ANOVAfor compressive strength and water absorption of the coated specimens.

	Factors	Pooling	DOF (f)	Sum of Squares (SS)	Variance (V)	F-Ratio (F)	Pure SS (SS’)	Percentage Contribution (P, %)
**compressive strength**	A	No	2	21.163	10.582	8.379	19.684	65.037
	B	No	2	4.051	2.026	1.604	2.572	8.498
	C	Yes	(2)	(1.479)	-	-	-	-
	Error	-	2	3.573	1.787	-	6.731	22.239
	Total	-	6	30.266	-	-	-	100
**water absorption**	A	No	2	0.522	0.261	16.473	0.519	57.654
	B	No	2	0.314	0.157	9.89	0.311	34.592
	C	Yes	(2)	(0.003)	-	-	-	-
	Error	-	2	0.06	0.03	-	0.07	7.754
	Total	-	6	0.899	-	-	-	0.1
**F**_0.01_(2,4)=18, **F**_0.05_(2,4)=6.94, **F**_0.10_(2,4)=4.32, **F**_0.25_(2,4)=2								
